# Global Motor System Suppression as the Primary Mechanism of Human Action Stopping: Challenging the Pause-Then-Cancel Model

**DOI:** 10.1523/JNEUROSCI.0447-25.2025

**Published:** 2025-08-01

**Authors:** Eashan Ray Chaudhuri, Tiombe Long, Ricci Hannah

**Affiliations:** Centre for Human & Applied Physiological Sciences, King’s College London, London SE1 1UL, United Kingdom

**Keywords:** electromyography, inhibitory control, motor cortex, response inhibition, transcranial magnetic stimulation

## Abstract

The ability to stop a planned or ongoing action is fundamental to inhibitory control. A recent theory proposes that stopping involves two distinct phases: an initial global suppression of motor activity (“pause”) followed by a selective cancelation of the targeted action. However, the necessity of a second “cancel” stage remains debated. We tested whether global suppression alone is sufficient to stop movement by analyzing electromyography from task-relevant agonist and antagonist muscles, alongside transcranial magnetic stimulation measures of global motor suppression from task-irrelevant muscles, during a stop signal task in adult human participants of both sexes. In Experiment 1, reanalysis of ballistic finger movements revealed that agonist muscle offset consistently preceded behavioral stopping, aligning with the time course of global suppression. In Experiment 2, we extended these findings to whole-arm reaching movements, demonstrating that global motor suppression persisted beyond the termination of muscle activity when stopping prevented movement initiation, or lasted up to that point, disengaging in time for antagonist activation to interrupt movements that had already begun. These findings challenge the pause-then-cancel model, instead supporting a single-stage global suppression framework. They also suggest that the global suppression mechanism is not a rigid, top–down stopping mechanism but rather part of a broader motor control system that flexibly adjusts movement commands based on task demands.

## Significance Statement

A recent theory suggests that stopping an action involves a two-stage process: first pausing movement execution and then selectively canceling specific motor commands. Our study challenges this view, demonstrating that global motor suppression alone is sufficient to terminate motor commands. Using electromyography and transcranial magnetic stimulation during two distinct motor tasks, we show that suppression fully curtails agonist motor commands before behavioral stopping, without requiring a distinct cancelation step. These findings reshape our understanding of action stopping and provide a foundation for exploring the pathophysiology of movement and neuropsychiatric disorders associated with impaired behavioral suppression.

## Introduction

Many of us can recall moments when we were about to act but refrained at the last moment, like suppressing an angry outburst or reaching for a pan on the stove before realizing it is hot. This process, known as action stopping, is believed to involve a prefrontal–basal ganglia–thalamocortical network (Jahanshahi et al., 2015; Hannah and Aron, 2021). While evidence supports this network, key questions remain about how it achieves movement suppression.

Traditionally, action stopping has been viewed as a single-stage process where a global suppression of motor activity, evidenced by a reduction in corticospinal excitability measured with transcranial magnetic stimulation (TMS) over task-irrelevant motor cortical representations (Badry et al., 2009; Greenhouse et al., 2012; Jana et al., 2020), is sufficient to terminate movement. This suppression is thought to be mediated by the subthalamic nucleus (STN; Wessel et al., 2016, 2022) and, in this framework, accounts for stopping without requiring additional processes (Hannah and Aron, 2021).

However, a competing proposal, the “pause-then-cancel” model, suggests stopping unfolds in two stages (Schmidt and Berke, 2017): a global “pause” that temporarily suppresses motor activity, followed by a more selective “cancel” process that targets specific movement commands. Here, the global suppression represents only the initial pause stage (Diesburg and Wessel, 2021).

Several human studies have tested predictions of the pause-then-cancel model, with some evidence supporting the involvement of distinct pause and cancel processes (Tatz et al., 2021, 2024; Wadsley et al., 2023; Hervault and Wessel, 2025; Wu et al., 2025). The model itself was originally developed to explain early STN activity observed in rodent studies, where activation begins within ∼15 ms of a stop cue and well before movement cessation (Schmidt et al., 2013). However, this early activation is not mirrored in humans or nonhuman primates, where STN activity typically emerges closer to the time of behavioral stopping (Bastin et al., 2014; Benis et al., 2016; Pasquereau and Turner, 2017). This discrepancy raises a question about whether such a two-stage process is truly necessary to explain human stopping.

A direct way to evaluate this question is to examine the suppression dynamics on trials where muscle activity is initiated but then interrupted before producing overt movement. Previous work suggests that the initial decline in muscle activity follows the global suppression and precedes the behavioral latency of stopping, the stop signal reaction time (SSRT; Raud and Huster, 2017; Hannah et al., 2020; Jana et al., 2020; Raud et al., 2022). Interestingly, recent work proposed that the P3 event-related potential, which emerges in scalp EEG around SSRT, might reflect the secondary “cancel” process (Hervault et al., 2025; Hervault and Wessel, 2025). However, if muscle activity is completely suppressed before SSRT and hence before typical P3 onset, it would suggest that global suppression alone is sufficient to terminate movement, undermining the need for a distinct cancelation stage.

Beyond this theoretical debate, a wider question remains: how does global suppression operate during real-world actions? Most stopping studies focus on simple, ballistic movements, like button presses or saccades (Greenhouse et al., 2012; Jana et al., 2020; Tatz et al., 2021, 2024), which offer little opportunity for interruption once movement has begun. However, many natural actions, such as reaching for a hot pan, unfold over longer durations, allowing for suppression both before and after onset. Such cases require both terminating agonist muscle activity and activating antagonist muscles to brake the movement (Kudo and Ohtsuki, 1998; Atsma et al., 2018). This raises a critical question: does the same global suppression mechanism, well documented in ballistic tasks, also operate when halting naturalistic, nonballistic movements that are already underway but not yet complete? If so, how does it avoid disrupting necessary antagonist activity required for braking?

To address these questions, we conducted two experiments investigating the temporal dynamics of motor suppression in relation to agonist and antagonist muscle activity during ballistic button-press and nonballistic whole-arm reaching versions of the stop signal task.

## Materials and Methods

### Participants

Healthy, adult, human volunteers provided written informed consent to participate in two experiments. The experiments were approved by the UCSD Institutional Review Board (protocol #171285) and the Research Ethics Committee at King's College London (HR/DP-21/22-31662).

#### Experiment 1

Eighteen participants (11 females; age 19 ± 0.4 years; 15 right-handed) were recruited as part of a separate study (Jana et al., 2020). In this study, we reanalyzed a subset of the data and present new findings. One person's data were excluded for poor behavioral performance.

#### Experiment 2

Twenty-two participants (seven females; age 23 ± 1 years; 19 right-handed). Two people's data were excluded for poor quality behavioral and/or electromyography (EMG) data. The sample size was based on our previous work noted above (Jana et al., 2020), which used a similar paradigm to test related hypotheses and demonstrated robust effects on both behavioural and neurophysiological measures (*n* = 17). This sample size also aligns with recent studies using similar methods to examine global motor suppression (Raud et al., 2020; Wadsley et al., 2023).

### Stop signal task

The tasks were coded using MATLAB (2016b and 2022b; MathWorks) in conjunction with Psychtoolbox (Brainard, 1997). Experiment 1, which has been described in detail previously (Jana et al., 2020), involved participants responding to white arrows on a screen by pressing keys with either their left index or pinky finger, depending on the arrow's direction. Participants had 1 s to make a response and were encouraged to respond as quickly and accurately as possible. Trials requiring a response, termed “go” trials, accounted for 75% of all trials. In the remaining 25% of trials, the arrow turned red after a variable delay (the stop signal delay, SSD), signaling participants to try to stop the impending movement. The SSD increased by 50 ms after a successful stop and decreased by 50 ms after a failed stop.

**Figure 1.**
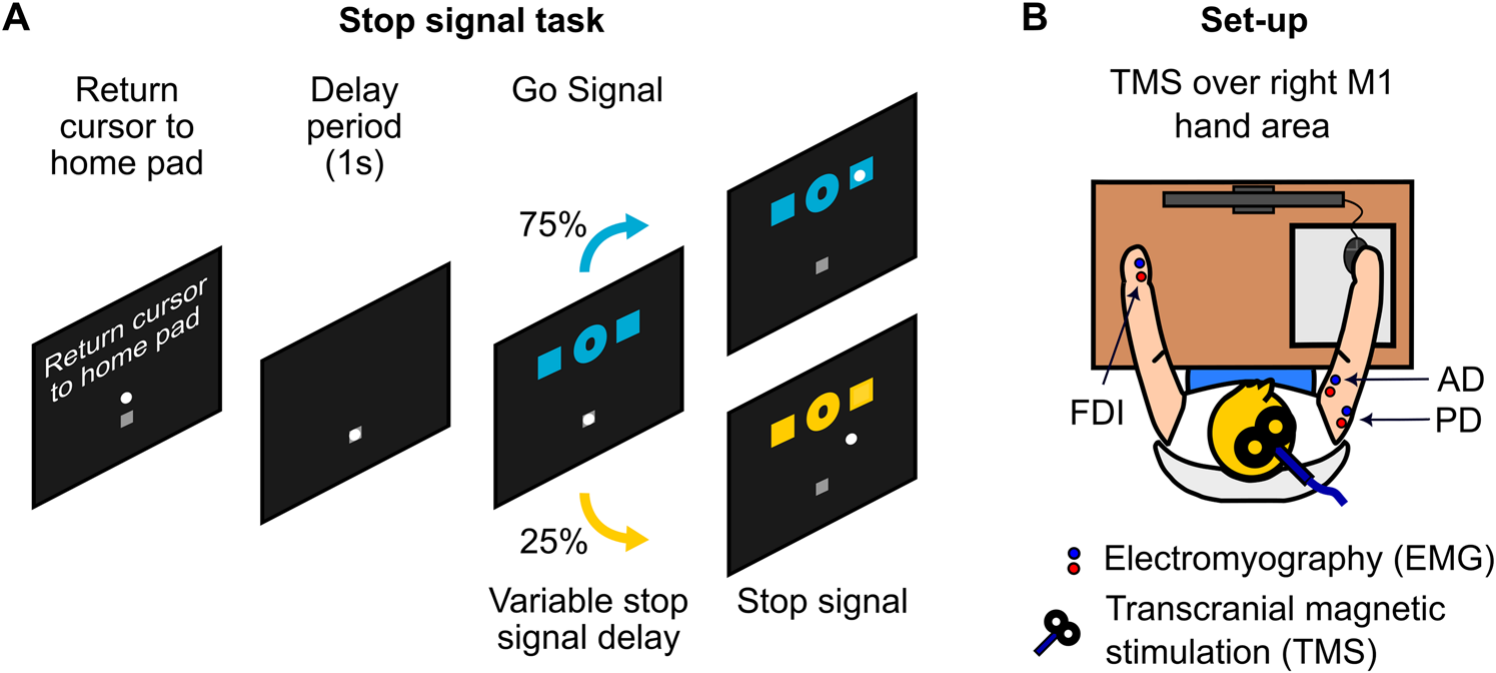
Experimental procedures and setup employed in Experiment 2. ***A***, Sequence of events in the whole-arm reaching stop signal task. Participants controlled a cursor using a computer mouse, starting each trial in the home pad before receiving a directional cue (“Q” for left, “O” for right) to move to one of two target squares. In go trials (75%), participants made the cued movement. In stop trials (25%), a stop signal (color change) instructed participants to stop the movement after a variable SSD. ***B***, Schematic of the experimental setup. Participants were seated in front of a screen, with EMG electrodes recording from the right anterior deltoid (AD, agonist), right posterior deltoid (PD, antagonist), and the left first dorsal interosseous (FDI, task-irrelevant muscle). A TMS coil over the right motor cortex elicited MEPs in the left FDI to assess global motor suppression, with TMS delivered at fixed time points relative to the stop signal or its yoked equivalent on go trials.

The task in Experiment 2 (Fig. 1*A*) built upon our recently developed whole-arm reaching version of the stop signal task (Hannah et al., 2023). In this version, participants moved a computer mouse cursor toward targets displayed on the screen. The sensitivity of the computer mouse was intentionally reduced so that participants were required to make larger, whole-arm movements, ∼10 cm displacement in the anterior–posterior plane, to reach the targets. Each trial began with participants positioning the cursor inside a designated small square, referred to as the “home pad.” One second after the cursor entered the home pad, two blue target squares and a go signal (a letter) appeared simultaneously on the screen. The letter “Q” instructed participants to move the cursor toward the left target, while “O” indicated movement toward the right target. These cues were intentionally chosen for their visual similarity, in order to slow response initiation and reduce the likelihood of SSD floor effects or violations of context independence (Bissett et al., 2021). Participants were instructed to execute their movements in a single, smooth motion, aiming for both speed and accuracy. Trials requiring a response were termed “go” trials. Failure to reach the target within the allotted time of 1.5 s resulted in a trial timeout, accompanied by a message indicating “too slow.”

As before, in a subset of trials (25%), a stop signal, indicated by a color change, was introduced following a variable SSD. Here, the SSD increased by ∼33.3 ms after a successful stop and decreased by ∼33.3 ms after a failed stop (Hannah et al., 2023). Participants were instructed to halt their movements promptly upon presentation of the stop signal. For the purposes of adaptive tracking, any movement that caused the mouse cursor to exit the home pad was classified as a response (i.e., a failed stop), whereas successful stopping was defined as the cursor remaining within the home pad.

However, in practice, participants were almost always able to interrupt the ongoing movement before it reached the target (∼96% stop trials; Hannah et al., 2023). This allowed us to study stopping at different points during movement execution: prior to, just after, and well after initiation. While this operational definition was useful for adaptive tracking, many of these so-called failed stops were in fact successful interruptions of action.

We therefore refer to these trials in Experiment 2 as “early stops” and “late stops” when discussing the data, to reflect the timing of inhibition within the movement rather than to imply failure or success in the conventional stop signal task sense. Importantly, although late stop trials involved partial movement, they were considered instances of successful stopping, as the action was canceled prior to reaching the target. Complete failures to reach interrupt the movement before the targets were termed “trigger failures,” assumed to represent failures to initiate the stop process altogether (Hannah et al., 2023).

At each block's end, participants received feedback on their average total response time. They were encouraged to speed their responses if they began to slow down or to exert their best effort to stop if stopping accuracy fell below 30%. Participants rested for as long as they needed between blocks.

### EMG

In Experiment 1, surface EMG signals were recorded from the left first dorsal interosseus (FDI) and abductor digiti minimi (ADM) muscles. Signals were bandpass amplified (×5,000; Grass QP511 AC amplifier, Grass Instruments) with a cutoff frequency ranging between 30 and 1,000 Hz. Signals were then digitized at 1,000 Hz (Micro 1401 mk II, Cambridge Electronic Design) and recorded via the data acquisition software (Signal version 4, Cambridge Electronic Design). Note that EMG was also recorded from the task-irrelevant extensor carpi radialis muscle to record motor-evoked potentials (MEPs) during the task. However, our focus here is on the task-relevant FDI and ADM muscles.

In Experiment 2, surface EMG was recorded from the anterior and posterior deltoid (AD and PD) muscles of the task-relevant right arm (Fig. 1*B*). These muscles served as agonists and antagonists in the reaching movement. EMG signals were obtained using a bipolar arrangement over the muscle, with a ground electrode situated over the acromion. Surface EMG was additionally recorded from the task-irrelevant FDI muscle of the left hand (Fig. 1*B*), which was not directly involved in the task. In each trial, signals were captured from 1 s preceding the go cue to 1.5 s following it.

EMG signals underwent bandpass amplification (×1,000; D360, Digitimer) with a frequency cutoff ranging from 10 to 2,000 Hz. Sampling occurred at a rate of 4 kHz using the Power 1401 mk II device (Cambridge Electronic Design). Data were acquired via the Signal v8 software (Cambridge Electronic Design).

### TMS

In Experiment 2, MEPs were evoked using a TMS device (Magstim 200^2^, The Magstim Company) delivering monophasic pulses and connected to a figure-of-eight coil (70 mm diameter, The Magstim Company). The coil was positioned on the scalp over the right primary motor cortex (M1) representation of the left FDI muscle and oriented so that the coil handle was approximately perpendicular to the central sulcus, that is, at ∼45° to the midsagittal line, and the initial phase of current induced in the brain was posterior-to-anterior across the central sulcus (Fig. 1*B*).

Prior to the experiments, the motor hot spot was determined as the position on the scalp where slightly suprathreshold stimuli produced the largest and most consistent MEPs in FDI. The position was marked on a cap worn by the participants. We then established the test stimulus intensity to be used during the task, which was set to produce a mean MEP amplitude of ∼1 mV while the participant was at rest.

MEPs were also evoked in the right AD muscle before beginning the main experiment to estimate the corticomuscular conduction time to the deltoid muscles. After determination of active motor threshold (AMT), 10 stimuli were delivered at 150% AMT during slight voluntary contraction (∼10% of maximum EMG).

### Procedure

In each experiment, participants began with a brief practice block to familiarize themselves with the task and to estimate an appropriate initial SSD for each individual. This titration was necessary because participants vary in their reaction times (RTs) and SSRTs, and the staircase algorithm used during the main task requires a starting SSD that is reasonably close to the value that yields ∼50% stopping accuracy.

Both Experiment 1 and 2 involved 12 blocks, each block consisting of 96 trials. During the task of Experiment 2, TMS was administered on both stop trials and on 50% of go trials. In Experiment 2, on five out of every six stop trials, a single TMS pulse at the test stimulus intensity was delivered at one of several time points: 100, 150, 200, 250, and 300 ms after the stop signal. In the remaining one out of six stop trials, no TMS was delivered. The latter helped minimize the total number of pulses administered. On go trials, TMS was synchronized with the timing of the stop signal on the preceding stop trial. Additionally, TMS was delivered at the time of the go signal, serving as a baseline estimate of excitability. Consequently, there were 48 trials per TMS time point on stop trials in Experiment 2.

### Data analysis

All analyses were performed using custom scripts written in MATLAB (2024b; MathWorks).

#### Behavioral performance

SSRT in both experiments was determined via the integration method (Verbruggen et al., 2019), which is based on the race model (Logan et al., 1984), hence termed SSRT_RM_. The model assumes a race between a go process and a stop process competing for completion. We also obtained SSRT estimates using a kinematic approach, identifying the timing of the peak in the cursor velocity-time curve in late stop trials as an estimate of when action stopping occurred (SSRT_K_; Hannah et al., 2023). Thus, SSRT_RM_ reflects stopping before detectable movement execution (early), whereas SSRT_K_ captures the interruption of an ongoing movement (late).

To derive SSRT_K_, we calculated resultant cursor displacement relative to the home pad, estimated resultant velocity, and measured the time of peak velocity on late stop trials relative to the stop signal to derive SSRT_K_. Unlike SSRT_RM_, SSRT_K_ does not rely on the assumptions of the independent race model (e.g., context independence or a single stopping process), offering a more direct, movement-based estimate of stopping latency. Its close correspondence with SSRT_RM_ across participants (see Results) supports the validity of both measures as behavioral indices of stopping despite their differing underlying assumptions.

In Experiment 2, go RTs were estimated as the time from the go signal to the time when the cursor first left the home pad, while total response times encompassed the time from the go to when the cursor entered the target.

Errors on go trials in Experiment 2 included omissions, missed targets, incorrect target hits, and false alarms. Kinematic data were also used to identify trials where the initial movement was in the wrong direction. Specifically, these errors were defined as trials where the cursor moved to the incorrect side of the midpoint of the screen for at least six consecutive frames (∼100 ms). These trials were classified as errors to avoid interpretive ambiguity, since suppressive mechanisms may contribute to the subsequent correction, potentially confounding the assessment of go trials. We also quantified trigger failures, reflecting failure to trigger the stop process, as the proportion of stop trials in which there was a complete failure to interrupt the movement before it reached the target (Hannah et al., 2023). This method has been previously validated against the Bayesian estimation of ex-Gaussian stop signal (BEESTS) model (Matzke et al., 2017).

#### EMG processing

EMG analysis followed a similar approach to our previous work (Hannah et al., 2020; Jana et al., 2020), with slight adjustments to the preprocessing. Data from Jana et al. (2020) in Experiment 1 were reanalyzed using the updated preprocessing method to ensure consistency across experiments.

In all cases, EMG signals were interpolated from −1 to 1.5 ms around the TMS to remove the stimulus artifact. The agonist and antagonist signals were then bandpass filtered between 10 and 2,000 Hz using a second-order Butterworth filter (roll-off 24 dB/octave) and bandstop filtered to remove mains hum (58–62 Hz for Experiment 1 and 48–52 Hz for Experiment 2). After filtering, signals were full-wave rectified, and the root mean square (RMS) was computed with a centered 20 ms window. Task-irrelevant FDI EMG data in Experiment 2 were bandpass filtered as above.

**Figure 2.**
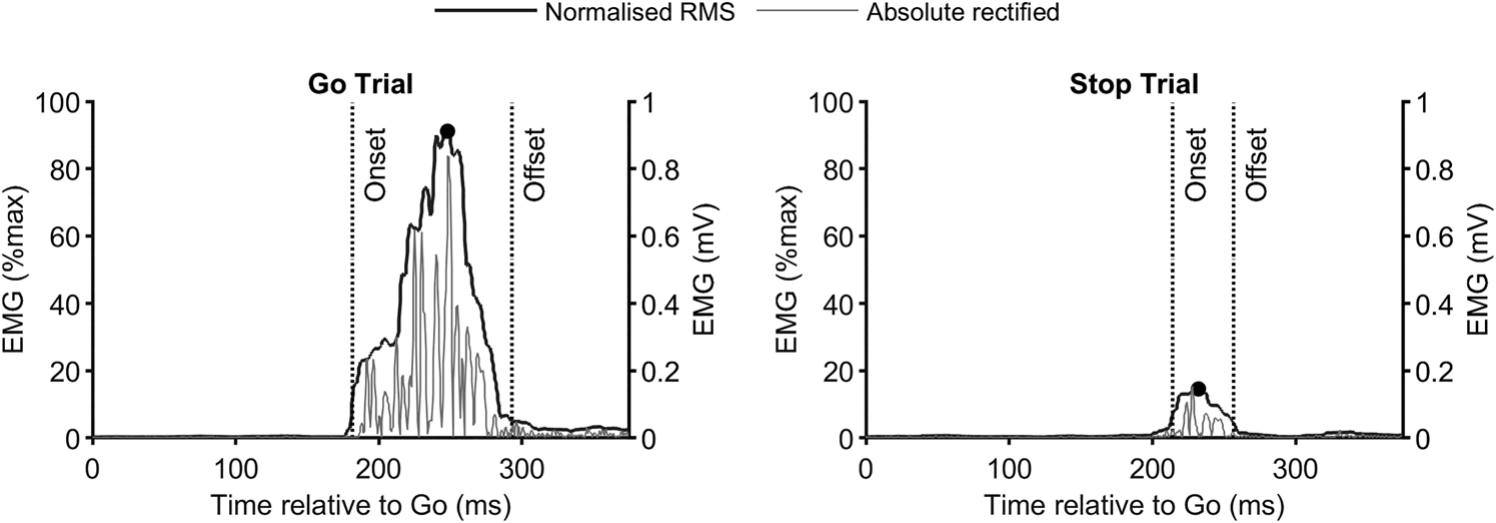
Representative data from a single participant showing normalized RMS EMG and absolute rectified EMG traces from the FDI muscle for single trials, along with the timing of EMG burst onsets, peaks (black circle), and offsets. ***A***, Go trial. ***B***, Stop trial. The minimal smoothing window applied to the RMS EMG provides reliable estimates of burst onsets, peaks, and offsets.

EMG bursts were identified as peaks exceeding 10% (Experiment 1) or 15% (Experiment 2) of the average peak EMG activity during correct go trials. A higher threshold was applied in Experiment 2 due to the increased variability and background activity associated with the reaching movement, where EMG activity supports both movement and limb stability at the start and end points. From each peak, the onset was determined by backtracking to the point where the signal fell below 5% (Experiment 1) or 10% (Experiment 2) of the peak for at least 20 consecutive milliseconds (Fig. 2*A*,*B*). Similarly, the offset was marked by tracking forward. The onset of EMG activity decline was simply defined as the time of the peak. These onset and offset times were computed on a per-trial basis and used for all timing analyses, including comparisons with SSRT and global suppression. Mean values were then calculated across trials for each participant. Outliers in EMG timing were identified as values exceeding 1.5 times the interquartile range of the first and third quartiles. Additionally, the algorithm occasionally captured early PD activity characterized by a slow rise beginning shortly after agonist onset (MacKinnon and Rothwell, 2000), rather than later burst-like activity triggered by the stop signal. Therefore, trials where PD onset occurred within 50 ms of the stop signal were not included in subsequent analyses.

Agonist muscle EMG amplitudes (FDI and ADM, Experiment 1; AD, Experiment 2) were normalized to the mean peak agonist activity in go trials (% max). For the antagonist PD muscle in Experiment 2, EMG amplitudes were normalized to the mean peak PD activity in late stop trials (% max).

In Experiment 1, we focused on the timing of both the decline and the offset of EMG activity in the agonist muscles relative to the stop signal and the behavioral latency of stopping, SSRT_RM_. Our primary aim was to demonstrate that agonist muscle activity terminated before the SSRT_RM_. Analyses were restricted to successful stop trials that contained suprathreshold EMG bursts. Trials without detectable EMG activity were excluded from these analyses.

Note that while EMG peaks (marking the onset of decline) are present in both go and stop trials, their characteristics differ systematically across conditions. In stop trials, the peak occurs earlier (*t*_(16)_ = 2.17; *p* = 0.0456; Cohen's *d* = 0.90), has a smaller amplitude (*t*_(16)_ = 22.87; *p* < 0.001; Cohen's *d* = 5.55), and marks the point at which EMG activity begins to decline rapidly, coinciding with the onset of global motor suppression (Jana et al., 2020). This suggests that the peak itself reflects the initiation of inhibition in stop trials and is informative about the timing of stopping.

In Experiment 2, we extended this investigation to the agonist AD and antagonist PD muscles. Specifically, we analyzed the timing of EMG decline and offset in the agonist muscle and the onset of EMG activity in the antagonist muscle, relative to the stop signal, SSRT_RM_ and SSRT_K­_, and the global suppression. The goals were to replicate Experiment 1's findings by confirming a rapid termination in agonist activity before the SSRT and to explore how this termination corresponded with the timing and duration of global suppression. Additionally, we investigated the relative timing of antagonist muscle onset and global suppression.

For Experiment 2, MEP amplitudes in the task-irrelevant left FDI muscle were measured trial-by-trial within a 30 ms window from 18 ms after the TMS. Data were included if FDI EMG activity in the 100 ms before the TMS was <0.05 mV. MEP amplitudes recorded at the go signal in go trials were averaged as a baseline for comparison across other TMS time points (100, 150, 200, 250, 300 ms after the stop signal). For each TMS time point, MEP data were averaged within each trial type (correct go, early stop, late stop) and expressed as a ratio relative to go signal MEP amplitudes.

#### Corticomuscular conduction time

The AD MEP onset latency across 10 trials was identified visually, and corticomuscular conduction time was taken as the minimum value across all stimuli (Hamada et al., 2013; Hannah and Rothwell, 2017).

### Statistical analyses

Data are presented as mean ± SEM. Statistical significance for all analyses was set at *p* < 0.05.

In Experiment 1, agonist EMG timings (peak, offset) were averaged across the FDI and ADM muscles and compared with the SSRT using paired-sample *t* tests. The relationship between EMG timings and SSRT were assessed using robust linear regression (“robustfit” function, MATLAB). Cohen's *d* was employed as an estimate of the effect size.

In Experiment 2, EMG timings for the agonist (peak, offset) and antagonist (onset) muscles were compared with SSRT using paired-sample *t* tests, and their relationships with SSRT were assessed using robust linear regression. A repeated-measure ANOVA was conducted to assess the effects of the trial type (go, early stop, late stop) and TMS time point (100–300 ms) on task-irrelevant FDI MEP amplitudes. Post hoc paired *t* tests were conducted to examine significant differences between trial types at each time point. Additional paired *t* tests compared MEP amplitudes at each time point and trial type to baseline MEP amplitudes to determine whether they were significantly below baseline. The similarity of SSRT_RM_ and SSRT_K_ was evaluated via paired *t* tests and robust linear regression, along with Bayes factor (BF_10_).

## Results

### Experiment 1

We reanalyzed EMG data from a previous study (Jana et al., 2020) to examine the timing of agonist EMG offset in relation to the SSRT_RM_.

As previously reported, participants exhibited typical stop signal task performance, with go RTs of 430 ± 17 ms, failed stop RTs of 391 ± 12 ms, mean SSD of 194 ± 18 ms, and pStop of 49 ± 1%. Mean failed stop RT was therefore shorter than go RT at the group level and in 15/17 individuals. Notably, small EMG bursts were present even in successful stop trials, suggesting that motor commands had been initiated but successfully curtailed before producing a behavioral response.

In our earlier work (Jana et al., 2020), we reported that the peak of agonist EMG (160 ± 9 ms), marking the onset of signal decline, occurred well before SSRT_RM_ (217 ± 6 ms) and shortly after the onset of global suppression. This temporal relationship suggested a link between suppression of the corticospinal pathway and EMG attenuation. However, that analysis focused on the onset of decline rather than complete suppression. Here, we directly quantified the time of EMG offset to assess when motor output is fully terminated.

**Figure 3.**
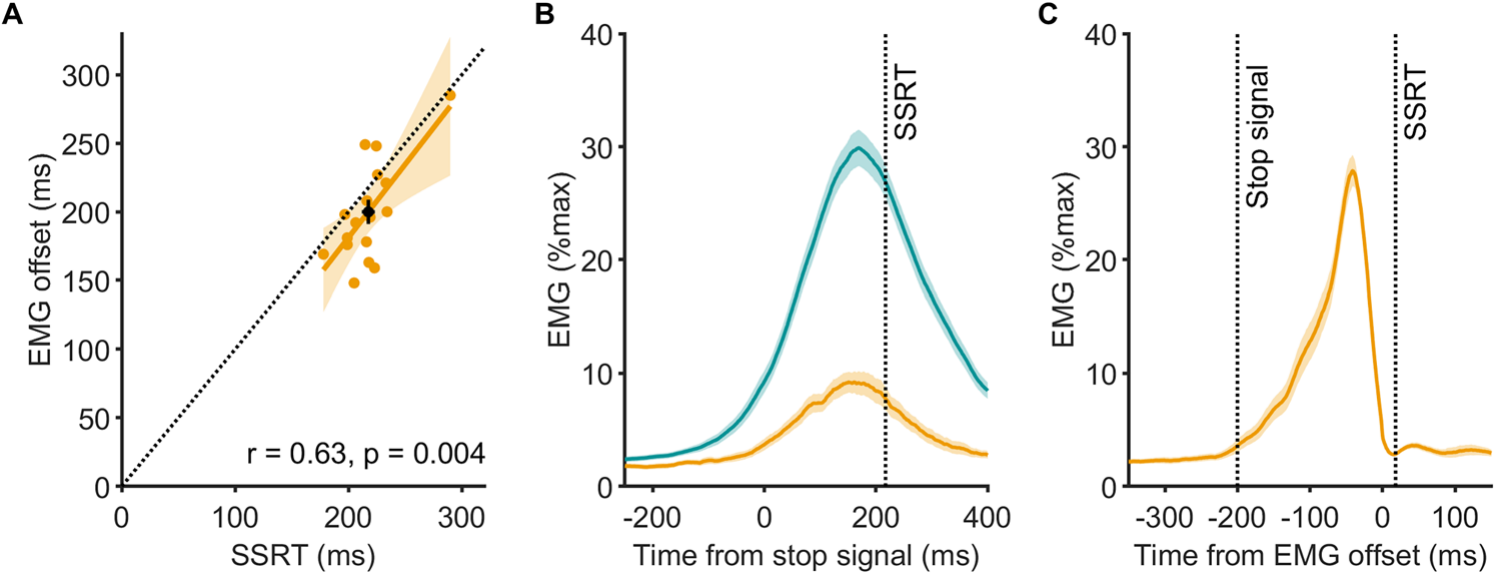
Timing and dynamics of agonist EMG suppression in successful stop trials (Experiment 1). ***A***, Scatterplot showing the relationship between SSRT_RM_ and agonist EMG offset time. The dashed line indicates unity, and the black circle marks the group mean ± SEM. Most individual data points (yellow circles) fall below the unity line, indicating that EMG offset occurred before behavioral stopping in the majority of participants. ***B***, Stop-aligned average EMG traces (FDI and ADM) for go and successful stop trials. Although partial bursts in successful stops are lower in amplitude and shorter in duration, the average waveform appears to decline gradually, seemingly extending beyond the SSRT_RM_. Shaded areas indicate SEM. ***C***, EMG traces aligned to offset reveal a sharper and more complete decline to near-baseline levels shortly after the burst peak. While not all trials showed EMG offset before SSRT_RM_, the average timing did, as reflected in the group mean and correlation in ***A***. The apparent persistence of EMG beyond SSRT in ***B*** arises from temporal smearing due to variability in EMG timing across trials and individuals. Offset alignment in panel ***C*** reduces this distortion and better visualizes the true average timing of muscle suppression. See Figures S1 and S2 for corresponding single-subject data aligned to the stop and the offset.

We estimated EMG offset on a trial-by-trial basis by applying a statistical threshold to each detected suprathreshold burst (Fig. 2). EMG bursts were present in 70 ± 4% of successful stop trials. The average burst amplitude reached 43 ± 2% of the go trial peak, indicating these were not marginal events. On average, the decline onset occurred at 155 ± 8 ms after the stop signal, and the offset followed at 201 ± 9 ms, 46 ± 4 ms later, and significantly earlier than SSRT_RM_ (mean difference, –17 ± 6; *t*_(16)_ = 2.59; *p* = 0.020; Cohen's *d* = 0.63). Thirteen out of seventeen participants show this pattern (Fig. 3*A*). Additionally, the EMG offset and SSRT_RM_ were positively correlated, reinforcing their close relationship (Fig. 3*A*).

Accounting for the corticomuscular conduction latency (23.3 ± 0.3 ms), we estimate that cortical commands ceased ∼177 ± 9 ms after the stop signal, 40 ± 7 ms before SSRT_RM_. Sixteen out of seventeen individuals exhibited this pattern. This timing fits within the window of global MEP suppression, which lasted until at least 180 ms poststop in the same participants (Jana et al., 2020), reinforcing the idea that global suppression and EMG termination are tightly coupled.

Although these trial-level data provide strong evidence that EMG activity is terminated prior to SSRT_RM_, group-averaged EMG traces aligned to the stop signal (Fig. 3*B*) appear to show EMG declining slowly, beyond SSRT. This apparent lag is a known artifact of temporal smearing: variability in EMG onset, peak, and offset across trials and individuals flattens the average waveform when aligned to an external event (Salomoni et al., 2025). For example, despite within-subject normalization to 100%, go trial EMG peaks at only ∼30% in the group average (Fig. 3*B*). A similar distortion is seen in successful stop trials (Fig. 3*B*) and is frequently present at the individual participant level in Figure S1.

Importantly, while EMG offset preceded SSRT_RM_ on average, not all trials or individuals exhibited this ordering. Across participants, almost two-thirds of successful stop trials (64 ± 4%) showed EMG offset prior to SSRT_RM_. When accounting for the corticomuscular conduction time (∼23 ms), which reflects the timing of cortical command termination, this proportion increased to 72 ± 4%. This variability likely contributes to the appearance of extended EMG activity in stop-aligned averages.

It is also important to note that SSRT_RM_ is estimated as a single value per participant, whereas the EMG offset is measured on a per-trial basis. As such, comparisons between the two inherently underestimate the proportion of trials where suppression occurs before stopping. If we could estimate SSRT on a single-trial basis or consider its distribution, a greater proportion of trials would likely show EMG offset preceding SSRT. For example, the standard deviation of SSRT estimated using the BEESTS model is ∼50 ms (Jana et al., 2020), which is comparable to the variability in EMG suppression onset latencies in our data (57 ± 7 ms). This interpretation is also supported by data from Experiment 2 (late stop trials), where SSRT was estimated at the single-trial level and EMG offset preceded SSRT_K_ in ∼90% of trials (see below).

To mitigate this smearing issue, we also present offset-aligned traces (Fig. 3*C*), where EMG is time-locked to its physiological endpoint. These plots reduce temporal smearing and provide a clearer visualization of the average timing of EMG suppression. This effect is further supported by individual offset-aligned traces shown in Figure S2, which demonstrate a sharper and more consistent return to the baseline across participants. These plots are not used in statistical analyses but help reconcile group-level visualizations with trial-level dynamics.

Together, these findings demonstrate that agonist EMG offset precedes behavioral stopping and is closely time-locked to the period of global motor suppression. To determine whether similar suppression dynamics extend to more complex, multijoint reaching movements, we conducted a second experiment with a longer measurement window (300 ms poststop) to track suppression and its interaction with antagonist recruitment.

### Experiment 2

#### Stop signal task performance

The probability of stopping (pStop) was ∼50% (Table 1), indicating effective implementation of the staircasing procedure. SSRT_RM_ (264 ms) fell within the typical range observed in behavioral stopping latency experiments across various effectors and devices (Brunamonti et al., 2012; Greenhouse et al., 2012; Atsma et al., 2018; Hannah et al., 2020 , 2023; Jana et al., 2020; Tatz et al., 2021). Importantly, the mean SSD was ∼253 ms, which lies outside the range where violations of context independence are problematic and interfere with estimates of SSRT_RM_ (Bissett et al., 2021). Furthermore, we included SSRT_K_, a kinematic estimate of movement interruption that avoids assumptions of the race model, to provide a complementary and model-agnostic stopping latency measure (see below).

**Table 1.** Stop signal task performance in Experiment 2 (*n* = 20)

Stop signal task indices	
Go RT (ms)	508 ± 19
Late stop RT (ms)	465 ± 14
SSD (ms)	253 ± 23
Probability of stopping (%)	49 ± 1
SSRT (ms)	264 ± 6
Go total response time (ms)	926 ± 26
Go errors	
Go total errors (%)	6.3 ± 1.0
Kinematic indices	
Go peak velocity (px/s)	3,184 ± 85
Late stop peak velocity (px/s)	1,826 ± 89
SSRT_K_ (ms)	272 ± 9
Trigger failures (%)	2.8 ± 1.3

Data are mean ± SEM.

The average go RT of ∼500 ms (Table 1) was within the expected range for stopping tasks (Brunamonti et al., 2012; Greenhouse et al., 2012; Atsma et al., 2018; Hannah et al., 2020, 2023; Jana et al., 2020; Tatz et al., 2021). The total response time, from the go signal to reaching the target, averaged 926 ms, implying a movement duration of ∼400 ms. As expected, late stop RT was shorter than Go RT at the group level, and in 19/20 participants, consistent with race model predictions (Logan et al., 1984; Verbruggen et al., 2019). The overall go trial error rate was acceptable (Table 1, 6.3%; range, 0.3–18.6%), with practically all errors being movements initially directed toward the wrong target (5.9%). Since such errors may involve suppressive mechanisms similar to stopping, these trials were excluded from analyses, including MEP comparisons.

**Figure 4.**
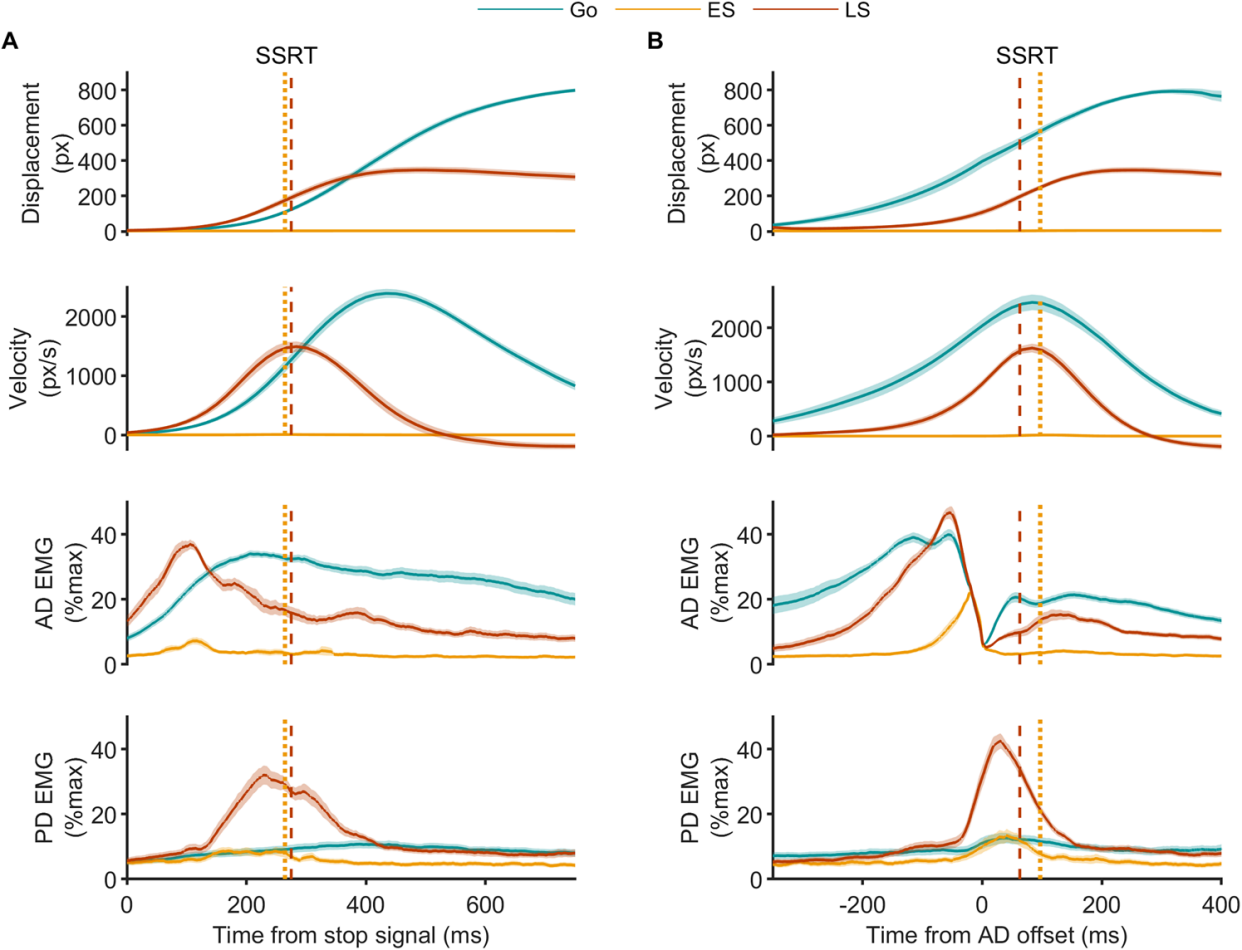
Group mean resultant cursor displacement and velocity, as well as EMG from the agonist (anterior deltoid, AD) and antagonist (posterior deltoid, PD) muscles during reaching movements in Experiment 2. ***A***, Data time-aligned to the stop signal on each trial or to the most recent SSD in go trials. Colored lines represent different trial types: go, early stop (ES), and late stop (LS). In late stop trials, displacement, velocity, and agonist activity are truncated relative to go trials, indicating interruption of an already-initiated movement. This is accompanied by a burst of antagonist activity, likely reflecting active braking. In contrast, early stops show minimal displacement and small, brief bursts of agonist or antagonist activity. Vertical lines indicate SSRT_RM_ (dotted) and SSRT_K_ (dashed). ***B***, Same data time-aligned to agonist offset. Agonist activity shows a rapid decline to baseline before SSRT_RM_ (early stop) and SSRT_K_ (late stop). Antagonist activity shows a sharp increase around agonist offset in late stop trials, preceding SSRT_K_. Following that, both late stop and go trials show a secondary agonist EMG burst, despite the absence of a stop signal in the latter, supporting the interpretation that this late activity reflects postural stabilization of the displaced arm rather than continued movement generation. Data are group mean ± SEM.

As is common in stop signal paradigms, participants likely engaged some degree of proactive control due to the anticipation of needing to stop. Despite this, reaching movements remained relatively fast, although not ballistic. The absence of a clear triphasic agonist–antagonist–agonist EMG pattern in go trials (Fig. 4*A*,*B*), a hallmark of ballistic movements (Brown and Gilleard, 1991), supports this interpretation. This movement profile was intentional, as it better reflects naturalistic reaching behaviors. Importantly, consistent agonist EMG bursts were still observed in ∼70% of stop trials (see below), in line with Experiment 1 and indicating that participants initiated a prepotent response requiring reactive stopping.

#### Movement kinematics and muscle activity during the stop signal task

##### Go trials: baseline kinematics and muscle activity

In go trials, the agonist (AD) muscle exhibited a rapid increase in activation before the movement onset, peaking prior to peak movement velocity (Fig. 4*A*,*B*). Antagonist (PD) EMG activity was detected in 57 ± 7% trials. The relative infrequency and small amplitude of antagonist EMG bursts (42 ± 4% of the average peak observed in late stop trials), as well as their variable timing across participants, made their presence less prominent in the averaged time-series data (Fig. 4*A*,*B*).

##### Early stop trials: absent movements and truncated muscle activity

As expected, early stop trials showed no overt movement, with cursor displacement restricted to the “home pad” (Fig. 4*A*,*B*). However, small bursts of agonist and antagonist activity were present in a subset of early stop trials (40 ± 5% and 47 ± 7% trials, respectively). The peak amplitude of agonist activity in early stops reached 17 ± 2% of the average go trial peak, while antagonist activity reached 27 ± 4% of the average peak observed in late stop trials. These low-amplitude, variably timed bursts were not apparent in the group-averaged traces time-locked to the stop signal (Fig. 4*A*), and often difficult to discern even in individual stop-aligned traces (Fig. S3). However, they became more apparent in offset-aligned averages (Fig. 4*B*; Fig. S4), highlighting the impact of temporal smearing across trials and individuals (Salomoni et al., 2025). This reinforces the importance of trial-level analyses and event-aligned measures such as EMG offset to accurately capture burst dynamics.

**Figure 5.**
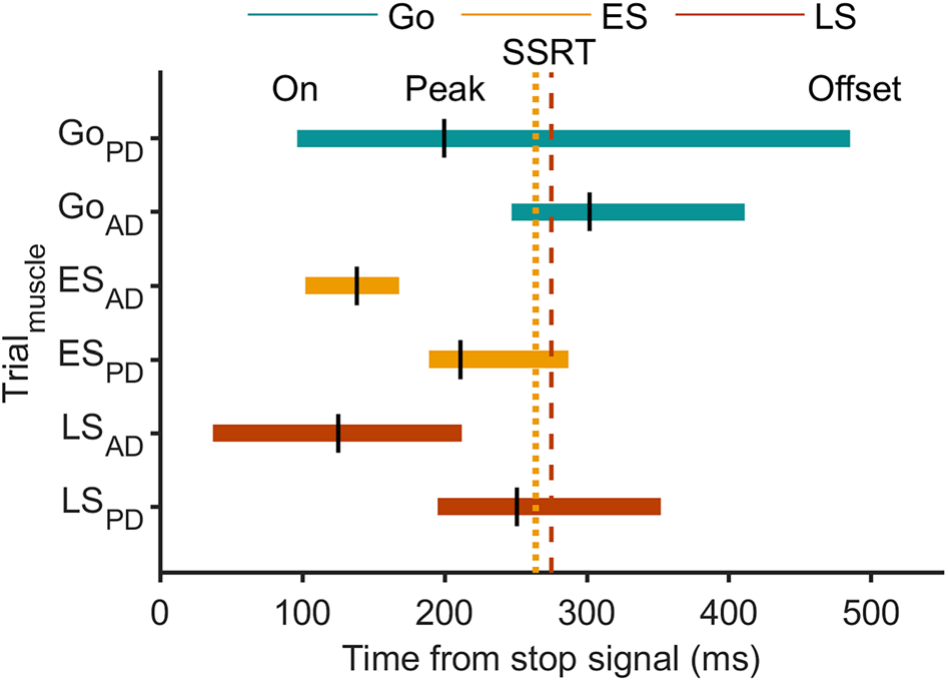
EMG burst timings with respect to the behavioral latency of stopping in Experiment 2. Timing of EMG bursts in the agonist (AD) and antagonist (PD) muscles relative to the stop signal. Colored horizontal bars represent the mean duration of muscle activity (onset to offset) across trial types, with vertical black lines indicating mean peak timing. The colored vertical lines mark the mean SSRT (dotted line, SSRT_RM_, dashed line, SSRT_K_). Data are group mean.

**Figure 6.**
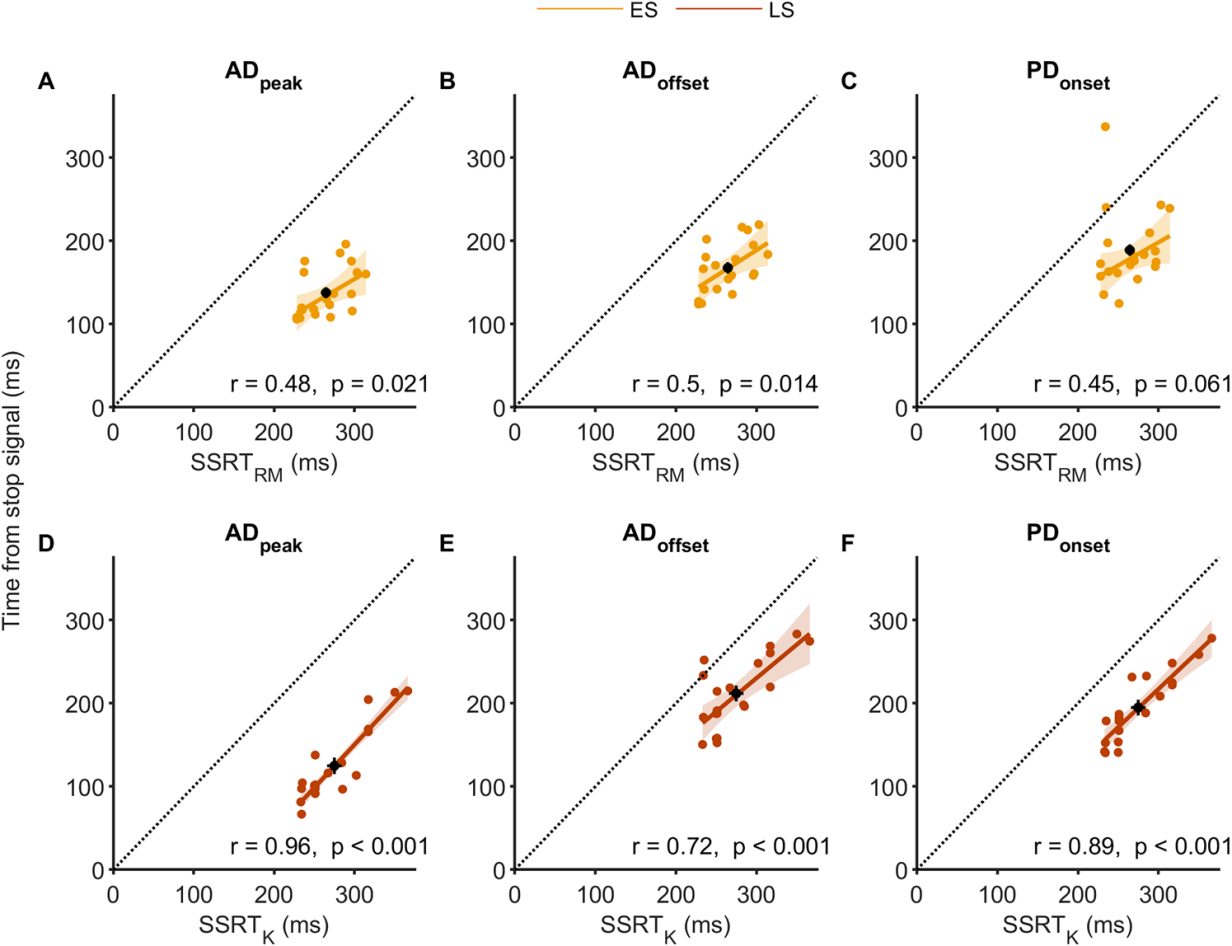
Relationships between stopping latencies (SSRT_RM_ and SSRT_K_) and EMG timings (agonist peak and offset, antagonist onset) across early (top row, ***A–C***) and late stop trials (bottom row, ***D–F***). Each panel plots individual participant data, with the dashed black line representing unity and the black circle indicating the group mean ± SEM for SSRT and the corresponding EMG timing. In nearly all cases, data points fall below the unity line, indicating that EMG events consistently occur prior to behavioral stopping. Moderate-to-strong correlations (except in panel ***C***) further support a systematic relationship of agonist EMG suppression and antagonist EMG activation dynamics with stopping latency.

The time from stop signal to peak agonist activity, marking the onset of the decline in EMG, in early stops was tightly constrained (138 ± 30 ms; Figs. 4*A*, 5, 6*A*). Agonist activity began to decline well before the SSRT_RM_ (Figs. 4*A*,*B*, 5, 6*A*). Crucially, agonist offset occurred at 168 ± 31 ms, just 30 ± 3 ms after peak activity, and significantly earlier than the SSRT_RM_ (mean difference, −97 ± 6 ms; *t*_(19)_ = 15.62; *p* = 2.7 × 10^12^; Cohen's *d* = 3.49; Figs. 4*B*, 5, 6*B*; and see Fig. S4 for individual participant data). Across participants, EMG offset preceded SSRT_RM_ in 88 ± 3% of early stop trials, further supporting the interpretation that motor commands were curtailed well in advance of behavioral stopping.

Despite the lack of overt movement, antagonist bursts were recruited shortly after agonist offset at an onset of 211 ± 7 ms with respect to the stop signal (Figs. 4*A*, 5, 6*C*). Antagonist onset also significantly preceded the SSRT (mean difference, −75 ± 12 ms; *t*_(19)_ = 6.39; *p* = 3.95 × 10^−6^; Cohen's *d* = 1.42; Figs. 4*A,B*, 5, 6*C*). Despite a trend toward a positive association between antagonist onset and SSRT in early stops the correlation was not statistically significant (Fig. 6*C*), potentially reflecting the lower reliability of antagonist recruitment in these trials, where movement was minimal.

##### Late stop trials: interrupted movements and coordinated agonist–antagonist dynamics

In late stop trials, movement was initiated but interrupted before reaching the target, as indicated by reduced peak velocity and displacement compared with go trials (Fig. 4*A*,*B*). Importantly, peak velocity occurred earlier in late stop trials compared with go trials relative to the stop signal (Fig. 4*A*). We interpret the timing of peak velocity in late stop trials as a behavioral marker of when the go-related motor command was curtailed (SSRT_K_), rather than when movement fully ceased. This better reflects the termination of descending drive, with subsequent motion continuing briefly due to mechanical inertia (see below for further discussion).

Our kinematic estimate of the latency of behavioral stopping, SSRT_K_ (272 ms; Table 1), was statistically similar to SSRT_RM_ (mean difference, 10 ± 6 ms; *t*_(19)_ = 1.61; *p* = 0.123; Cohen's *d* = 0.36; BF_10_ = 0.70 in favor of null hypothesis), and the two were strongly correlated (*r* = 0.70; *p* < 0.001), suggestive of a shared underlying mechanism (Hannah et al., 2023).

Agonist activation began earlier in late stop trials, consistent with shorter RTs in these trials (Fig. 4*A*). The agonist burst peaked earlier, had a smaller amplitude (84 ± 2% of the average go trial maximum), and declined more rapidly than in go trials (Fig. 4*A*). The time from stop signal to peak agonist activity, marking the onset of agonist decline, occurred within 125 ± 45 ms (Figs. 4*B*, 5, 6*D*). Agonist offset (212 ± 42 ms) followed shortly after the peak, again occurring significantly earlier than the SSRT_K_ (mean difference, −63 ± 7 ms; *t*_(19)_ = 9.08; *p* = 2.4 × 10^−8^; Cohen's *d* = 2.03; Figs. 4*B*, 5, 6*E*). Importantly, faster agonist offset was associated with shorter SSRT_K_ (Fig. 6*E*), reinforcing the link between early suppression at the muscle level and successful stopping. Given that the corticomuscular conduction time to the AD muscle was 9.6 ± 0.2 ms, the actual cessation of cortical commands must have occurred at ∼202 ms after the stop signal and therefore well before the SSRT_K_. Agonist EMG offset preceded SSRT_K_, measured at the single-trial level, in 88 ± 3% of late stop trials. Note, this estimate may be slightly conservative due to the presence of a second agonist burst, which could affect the algorithmic detection of the first burst's offset.

Following this initial offset, agonist EMG reemerged later in the trial (Fig. 4*B*). In stop-aligned group averages (Fig. 4*A*), this appeared as a single continuous burst with a gradual decline. However, Figure S5 reveals heterogeneity: some participants mirrored this pattern, while others showed clear suppression before SSRT and a distinct second burst beginning after.

Offset-aligned traces (Fig. 4*B*; Fig. S6) clarify this biphasic structure. In most individuals, EMG dropped below the baseline after the first burst, before reemerging post-SSRT. This second phase began during deceleration and extended beyond movement offset, suggesting it reflects postural support rather than continued movement. Its presence was graded—absent in early stops, moderate in late stops, and largest in go trials—consistent with increased stabilization demands for larger displacements. Figure S7 further supports out interpretation. In a single-participant exploratory experiment, agonist EMG amplitude during a 1.5 s postreach hold phase scaled systematically with reach displacement. This relationship suggests that the postreach EMG reflects the level of postural support required to maintain the limb's final position.

Antagonist bursts were present in nearly all late stop trials (93 ± 1%; Fig. 4*A*,*B*) and typically began after the initial agonist burst offset, at 195 ± 9 ms after the stop signal, but prior to SSRT_K_ (mean difference vs SSRT_K_, −80 ± 5 ms; *t*_(19)_ = 19.43; *p* = 5.3 × 10^−14^; Cohen's *d* = 4.34; Figs. 4, 5), and prior to the second bout of agonist activity. This temporal relationship suggests that SSRT_K_ marks a biomechanical transition: agonist-driven acceleration ceases, and antagonist-mediated braking begins. Earlier antagonist onset was also associated with shorter SSRTs (Fig. 6*F*), further supporting its role in halting movement.

The pattern of antagonist recruitment is consistent with prior action-stopping studies involving dynamic upper-limb and head movements (Kudo and Ohtsuki, 1998; Goonetilleke et al., 2010; Corneil et al., 2013; Atsma et al., 2018). In contrast, a static pressing task showed minimal antagonist activity (Van Boxtel et al., 2001), likely due to the absence of limb movement. In our data, antagonist burst amplitude scaled with the demands of stopping (i.e., late vs early stops; Fig. 4*B*), further supporting a reactive braking role. Together, these results support the interpretation that antagonist engagement in our task reflects a reactive braking response.

#### Global suppression and its timing relative to EMG activity

**Figure 7.**
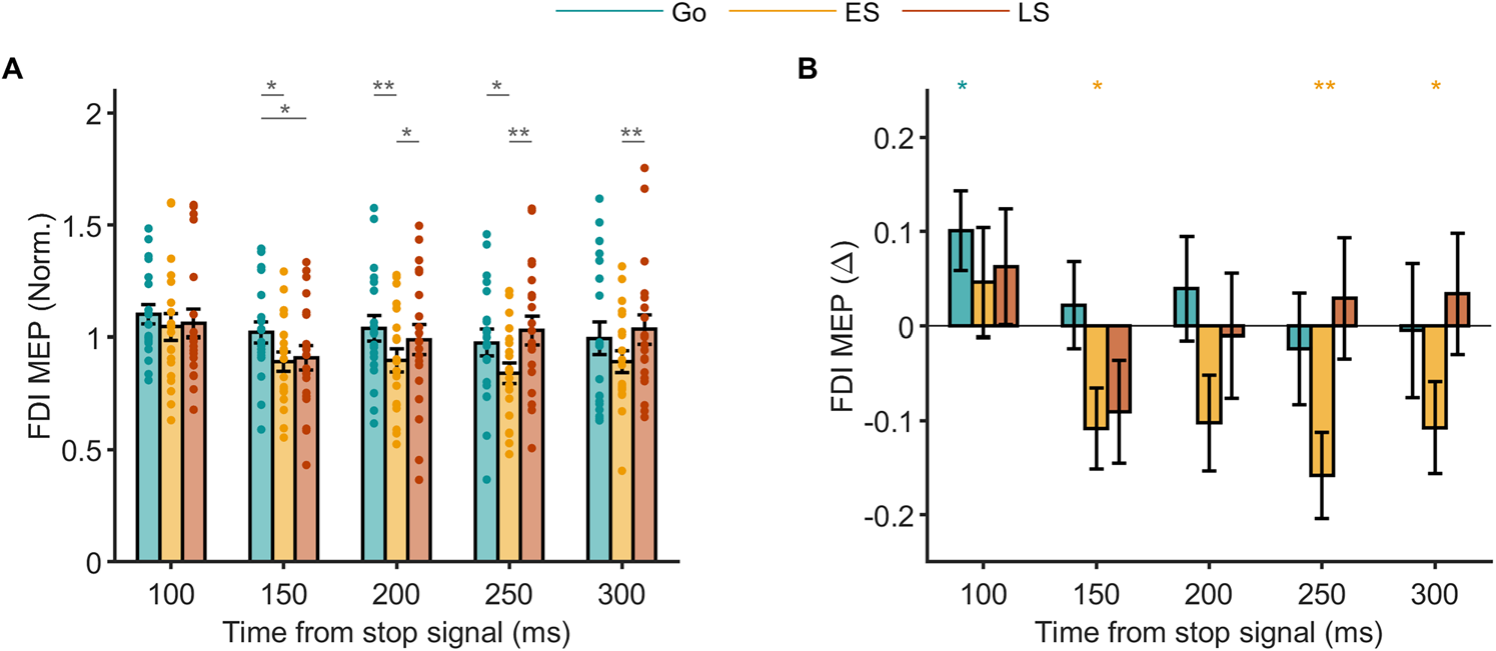
Time course of global suppression Experiment 2. ***A***, MEP amplitudes across go, early stop, and late stop trials in the task-irrelevant FDI normalized to those at the go signal (baseline). Data are group mean ± SEM with smaller symbols reflecting data from individual participants. Stars and associated horizontal bars indicate significant differences between trial types at each time point (*p* value: *p < 0.05; **p < 0.01; ***p < 0.001). ***B***, The same data expressed as a change for each trial type relative to the baseline. Stars indicate significant differences compared with baseline for each trial type (*p* value: *p < 0.05; **p < 0.01; ***p < 0.001).

In go trials, MEP amplitudes in the task-irrelevant FDI muscle remained stable across all time points, except for a significant elevation relative to the baseline at ∼100 ms after the stop signal (Fig. 7*A*,*B*).

In early stop trials, MEP amplitudes were significantly reduced compared with go trials between 150 and 250 ms (Fig. 7*A*) and were notably below the baseline at 150, 250, and 300 ms (Fig. 7*B*). These findings align with prior work reporting global MEP suppression starting within 140 ms of a stop signal (Jana et al., 2020).

In late stop trials, MEP amplitudes briefly decreased within 150 ms, exhibiting an initial suppression similar to early stops before returning to the baseline by 200 ms (Fig. 7*A*,*B*). This reduction was statistically significant relative to go trials. The pattern indicates that global suppression is transiently engaged and then rapidly withdrawn as the movement is interrupted.

Repeated-measure ANOVA on the normalized MEP data supported these findings, confirming a significant trial type × time interaction (*F*_(8,152)_ = 2.286; *p* = 0.024) and showing that corticospinal excitability varied across trial types over time. There was a main effect of time (*F*_(4,76)_ = 9.136; *p* = 4.42 × 10^−6^) but no effect of the trial type [*F*_(2,38)_ = 2.21, *p* = 0.123].

In summary, global suppression began ∼150 ms poststop signal, broadly aligning with the decline of agonist EMG in both early and late stop trials. In early stops, suppression persisted up to 300 ms after the stop signal, extending beyond agonist termination (∼200 ms) and SSRT (∼260 ms), overlapping with relatively weak and infrequent antagonist recruitment (∼200 ms). In late stops, suppression was shorter-lived, returning to the baseline by ∼200 ms, coinciding with agonist offset and onset of strong antagonist recruitment. These findings support a model where global suppression, agonist cancelation, and antagonist activation are temporally coordinated to either prevent or interrupt movement effectively.

## Discussion

To better understand how action stopping unfolds across the motor system, we examined the timing of agonist suppression, global motor suppression, and antagonist recruitment across two experiments.

### Global suppression halts agonist activity

In Experiment 1, agonist EMG bursts on successful stop trials were fully terminated ∼17 ms before the SSRT, with cortical motor commands likely ceasing ∼40 ms earlier, providing clear physiological evidence that motor commands are halted well before behavioral stopping. Experiment 2 extended these findings to nonballistic reaching movements. Agonist bursts were abruptly truncated, whether the movement was canceled before initiation or interrupted midexecution. In both cases, EMG offset preceded SSRT by ∼60–100 ms. We also found that global MEP suppression began around the same time as agonist EMG suppression and persisted until complete cessation of the initial agonist burst. This temporal alignment supports the view that global suppression is the primary mechanism responsible for canceling motor output (see “Postural goals and the secondary agonist bout” section for discussion of the secondary agonist bout in Experiment 2).

### Integration of global suppression and antagonist recruitment

The pattern of antagonist recruitment in our study highlights how action stopping integrates both neural suppression and biomechanical braking. In go trials, antagonist bursts were weak and inconsistent, suggesting they were not part of a strongly preprogrammed pattern (Brown and Gilleard, 1991). Late stop trials exhibited prominent antagonist bursts following agonist offset, reflecting a reactive response to arrest ongoing movement rather than a feedforward component of the original motor plan. Notably, global motor suppression subsided around the time of agonist termination and antagonist onset. This temporal coordination suggests a transition from neural suppression to active deceleration. In early stop trials, antagonist bursts were smaller and more variable, and global suppression persisted despite their presence, suggesting that antagonist activity alone is insufficient to terminate global suppression, especially in the absence of overt movement.

One explanation is that the motor system monitors predicted versus actual sensory consequences of movement (Wolpert and Flanagan, 2001), using this information to update control in real time. In late stops, sensory feedback from movement may trigger the shift from suppression to braking. In early stop trials, where feedback is minimal, suppression may persist as a precaution.

Although our primary focus is on cortical and corticospinal processes, this interpretation aligns with the cerebellum's role in predicting sensory outcomes (Tseng et al., 2007; Schlerf et al., 2012; Hull, 2020) and initiating antagonist bursts during braking (Hallett et al., 1975; Flament and Hore, 1986). The delayed withdrawal of global suppression relative to antagonist recruitment in early stops suggests that while M1 may contribute to antagonist activation, it is not essential. Subcortical structures like the cerebellum may trigger antagonist bursts, with M1 providing additional support, consistent with its role in triphasic activation patterns typical of ballistic movements (MacKinnon and Rothwell, 2000; Irlbacher et al., 2006).

While the global suppression appears to serve as the primary inhibitory mechanism, reciprocal inhibitory circuits within both cortical (Griffin and Strick, 2020) and spinal cord circuits (Vallbo et al., 1979; Day et al., 1984) may still support agonist suppression. These pathways could enable a smooth shift from suppression to braking without requiring a separate cancelation stage. Classic work shows that antagonist bursts are often preceded by agonist suppression (Hufschmidt and Hufschmidt, 1954), further supporting this possibility.

### Postural goals and the secondary agonist bout

To interpret the secondary agonist bout in late stop trials, we considered two possibilities: residual drive from the initial command versus a separate postural stabilization command. The first suggests lingering output from the original movement command, persisting after a brief pause. However, we argue it is better understood as a postural command rather than evidence of incomplete suppression. This bout emerged after the initial reach was canceled and persisted even after the limb came to rest, supporting a stabilizing role. Its presence in go trials and absence in early stops further suggest it reflects biomechanical demands rather than stopping-specific control.

Combined with the timing of global suppression and antagonist braking, these findings suggest a coordinated transition in motor control: global suppression halts the initial movement command, antagonist activity counters inertia, and the secondary agonist activity stabilizes the limb. While this sequence unfolds over time, it does not reflect multiple stages of neural suppression targeting the original command, as proposed in the pause-then-cancel model (Schmidt and Berke, 2017). Instead, the later components arise from the biomechanical demands of halting and stabilizing the body.

This pattern mirrors well-established agonist–antagonist dynamics observed during rapid, unperturbed reaching and single-joint movements without stop signals (Flanders and Herrmann, 1992; Buneo et al., 1994; Berardelli et al., 1996): a phasic burst in the agonist initiates movement, a brief pause allows antagonist braking, and a tonic agonist bout maintains posture against gravity. Crucially, prior work also showed that the two agonist bouts are functionally distinct: the initial phasic burst scales with movement speed, while the tonic bout does not (Flanders and Herrmann, 1992).

### Implications for theories of action stopping

The “pause-then-cancel” model posits that a secondary cancelation process follows an initial pause (Schmidt and Berke, 2017; Diesburg and Wessel, 2021). This is partly based on interpretations that stop-aligned average EMG offset lags behind SSRT, implying a second stage is needed to complete stopping (Hervault et al., 2025). However, our single-trial analyses suggest that agonist EMG termination occurs rapidly, before SSRT and within the period of global motor suppression. This suggests that a single, rapidly engaged suppressive mechanism can account for movement cancelation without invoking a distinct later-stage suppression process.

While we cannot rule out contributions from other basal ganglia structures, such as globus pallidus or striatum, as predicted by the pause-then-cancel model, their dynamics in humans remain largely uncharacterized. In contrast, single-unit recordings from the STN in humans (Benis et al., 2016) and nonhuman primates (Pasquereau and Turner, 2017) show activity peaking ∼130–170 ms after the stop signal, just prior to changes in EMG and SSRT, and closely aligned with the timing of agonist suppression in our data. Given the rapid suppression of agonist EMG in our experiments, any distinct cancel process would need to operate within a very narrow window to meaningfully influence termination.

In the absence of direct human evidence for a temporally distinct subcortical cancel mechanism beyond STN-mediated suppression, we interpret rapid EMG offset, closely aligned with global suppression, as a sufficient physiological marker of stopping. Future work may clarify whether pallido–striatal circuits in humans play an additional, functionally distinct role.

### Methodological considerations

The EMG burst detection rate in Experiment 1 (∼70%) was relatively high but within the range reported in a recent meta-analysis (Raud et al., 2022). This is likely due to the relatively fast RTs in our task, consistent with findings that burst detection rates negatively correlate with RTs (Raud et al., 2022). Importantly, burst frequency is unrelated to stopping latency (Raud et al., 2022), suggesting our elevated detection rate does not bias suppression timing estimates. Moreover, bursts in stop trials exceeded 40% (Experiment 1) and 80% (Experiment 2) of the go trial peak, indicating suppression timing was not driven by low-amplitude or borderline events.

Although stop-aligned average EMG appears to show delayed termination relative to SSRT, this reflects temporal smearing due to variability across trials and participants. Crucially, all timing analyses were based on single-trial EMG offsets, which consistently occurred before SSRT on average.

Race model-derived SSRTs depend on assumptions such as context independence (Bissett et al., 2021) and the absence of trigger failures (Matzke et al., 2017), which, if not met, can bias comparisons with physiological data. We therefore also included a model-free kinematic estimate (SSRT_K_), which yielded similar results and revealed low trigger failure rates. Our key findings held across experiments, effectors, and stopping indices and were supported by the consistent observation that agonist EMG was rapidly suppressed during the global motor suppression.

We defined kinematic stopping as the point of velocity reversal, which closely follows agonist EMG offset and coincides with peak antagonist activity, and therefore marks the transition from acceleration to deceleration. Later phases, e.g., the point of movement cessation, likely reflect electromechanical delays and inertia rather than active inhibitory control, making velocity reversal a more meaningful marker of stopping.

Finally, although our task required outright movement cancelation, many real-world scenarios demand redirection or reaiming. Future work should investigate how global suppression interacts with flexible goal updating to better define its role in adaptive motor control beyond stopping.

## Conclusion

Our findings challenge the pause-then-cancel model of action stopping by showing that agonist activity is abruptly terminated in synchrony with global motor suppression and before behavioral stopping, eliminating the need for a second process. Additionally, global motor suppression is dynamically coordinated with antagonist recruitment rather than acting as a rigid mechanism, highlighting its integration within broader motor control.

## Data Availability

Data and code are available upon request.
